# A Valepotriate Fraction of *Valeriana glechomifolia* Shows Sedative and Anxiolytic Properties and Impairs Recognition But Not Aversive Memory in Mice

**DOI:** 10.1093/ecam/nep232

**Published:** 2011-01-12

**Authors:** Natasha Maurmann, Gustavo Kellermann Reolon, Sandra Beatriz Rech, Arthur Germano Fett-Neto, Rafael Roesler

**Affiliations:** ^1^Graduate Program in Cellular and Molecular Biology, Center for Biotechnology, Federal University of Rio Grande do Sul, 91501 970 Porto Alegre, RS, Brazil; ^2^Laboratory of Molecular Neuropharmacology, Department of Pharmacology, Institute for Basic Health Sciences, Federal University of Rio Grande do Sul, Porto Alegre, Brazil; ^3^Faculty of Pharmacy, Federal University of Rio Grande do Sul, Porto Alegre, Brazil

## Abstract

Plants of the genus *Valeriana* (Valerianaceae) are used in traditional medicine as a mild sedative, antispasmodic and tranquilizer in many countries. This study was undertaken to explore the neurobehavioral effects of systemic administration of a valepotriate extract fraction of known quantitative composition of *Valeriana glechomifolia* (endemic of southern Brazil) in mice. Adult animals were treated with a single intraperitoneal injection of valepotriate fraction (VF) in the concentrations of 1, 3 or 10 mg kg^−1^, or with vehicle in the pre-training period before each behavioral test. During the exploration of an open field, mice treated with 10 mg kg^−1^ of VF showed reduced locomotion and exploratory behavior. Although overall habituation sessions for locomotion and exploratory behavior among vehicle control and doses of VF were not affected, comparison between open-field and habituation sessions within each treatment showed that VF administration at 1 and 10 mg kg^−1^ impaired habituation. In the elevated plus-maze test, mice treated with VF (10 mg kg^−1^) showed a significant increase in the percentage of time spent in the open arms without significant effects in the number of total arm entries. VF at 3 mg kg^−1^ produced an impairment of novel-object recognition memory. In contrast, VF did not affect fear-related memory assessed in an inhibitory avoidance task. The results indicate that VF can have sedative effects and affect behavioral parameters related to recognition memory.

## 1. Introduction

Complementary and alternative medicines (CAMs) have a long history of use for the treatment of sleep disorders [1]. The species of *Valeriana* are known for medical properties that date back to many centuries [2], and the herbal supplement valerian (*Valeriana officinalis*) is one of the most popular CAM therapies for insomnia [3, 4].

More than 100 constituents have been identified in *Valeriana* sp., including sesqui and monoterpenes (hydrophilic valerenic acids and the hydrophobic valepotriates, resp.), which may account for the activity in the *central nervous system* [2]. Valepotriates are iridoids with a cyclopenta(*c*)pyranoid skeleton, an epoxy ring and three ester linkages, without glycosidic linkages [5], and controversial pharmacological activity [6]; their degradation products, valtroxal, 8,9-didehydro-7-hydroxy-dolichodial, 11-ethoxyviburtinal and baldrinal may account for valerian's effect [7]. Although several clinical studies revealed sleep-improvement properties, there is no scientific agreement on the sedating mode of action or the active constituents responsible for the valerian effects [2, 3, 8, 9].

*Valeriana glechomifolia* Meyer is an herb that grows in a restricted area of southern Brazil, and is not currently used as a phytomedicine. This plant accumulates valepotriates, both in shoots (1.57 g% DW) and roots (0.47 g% DW) [10]. As an alternative to the extraction of field-grown plants of *V. glechomifolia* for studies on pharmacological properties of the species, we developed *in vitro* propagation protocols and studied the kinetics of growth and valepotriate production in aseptically cultivated plants [11–13].

The activity of valepotriates in the central nervous system remains inconclusive and the pharmacological effects of *V. glechomifolia* have not been examined yet. Therefore, a detailed behavioral and memory analysis of the effect of a valepotriate extract fraction of known quantitative composition from this species was carried out in mice model to evaluate locomotion (open field), anxiety (elevated plus maze), aversive memory (inhibitory avoidance) and declarative memory (object recognition).

## 2. Methods

### 2.1. Plant Material

*Valeriana glechomifolia* Meyer plants were collected in the region of Aparados da Serra, near the city of São Jose dos Ausentes (28°44′54′′ south and 50°03′57′′ west), state of Rio Grande do Sul, Brazil, in the autumn. The species was identified by Dr M. Sobral and a voucher specimen (Sobral, 7733) is deposited at the Herbarium of the Federal University of Rio Grande do Sul (ICN). The plants were frozen, lyophilized, powdered and stored in freezer.

### 2.2. Preparation of Chloroform Extract of Valerian

Lyophilized shoots and roots of the plant were crushed to a particle size <850 *μ*m. Approximately 100 g (dry weight) was extracted twice with 500 mL of chloroform for 15 min using a sonication bath (Ultrasonic). The extract was filtered through a glass filter and evaporated to dryness in vacuum at 40°C, yielding 4.21 g of extract.

### 2.3. Preparation of Semi-Purified Valepotriate Fraction

To further purify the valepotriate fraction (VF), the dried extract was separated by silica gel vacuum column chromatography with a hexane:chloroform gradient. The fraction containing valepotriates was monitored by preparative thin-layer chromatography (TLC) with chloroform : methanol (50 : 0.5) as eluent [10]. The VF was concentrated and used in the behavior tests.

### 2.4. Quantification of Valepotriates

High-pressure liquid chromatography (HPLC) analysis of valepotriates in the VF was performed as previously described [10]. The VF (3 × 5 mg) was dissolved in methanol and analyzed in a Shimadzu equipment, using a Nova-Pack C18 column and pre-column (Waters). The mobile phase was acetonitrile/water 50 : 50 (v/v), and the flow rate 1 mL min^−1^; detection was done at 208 nm (didrovaltrate, retention time of 19.8 min) and 254 nm (acevaltrate, retention time of 18.1 min and valtrate, 34.8 min) [10–14]. The valepotriates used as external standards were isolated as described elsewhere [10] and the identity and purity of the compounds were confirmed by nuclear magnetic resonance (^1^H NMR) [14]. The spectral data were identical to those reported in the literature [15]. The phytochemical analysis revealed that the VF contained 96% of valepotriates (5 mg of VF contained 2.05 ± 0.11 mg of didrovaltrate, 1.66 ± 0.05 mg of valtrate and 1.10 ± 0.01 mg of acevaltrate).

### 2.5. Animals

Swiss male CF1 mice (60–90 days old with mean body weight of 36.18 ± 3.41 g) obtained from the State Foundation for Production and Research in Health (FEPPS), Porto Alegre/Rio Grande do Sul/Brazil, were used in the pharmacological assays. Each group consisted of 8–10 animals, kept on a 12 h light/dark cycle with food and water available *ad libitum*. Behavioral procedures were conducted between 11 a.m. and 6 p.m. Experimental procedures were performed in accordance with the European Convention for the Protection of Vertebrate Animals Used for Experimental and Other Scientific Purposes (European Treaty Series—No. 170 revised 2005) and the procedures of the Brazilian College of Laboratory Animals (COBEA). The experimental protocols were approved by the institutional research ethics and animal care committee (document number GPPG-HCPA 05–519). All efforts were made to minimize the number of animals and their suffering.

### 2.6. Drugs and Pharmacological Procedures

The VF was suspended in saline with Tween-80, 5.0% (v/v). Fresh solutions were prepared each time and intraperitoneally injected in a volume of 10 mL kg^−1^ body weight at the doses of 1, 3 or 10 mg kg^−1^; the control mice were injected with vehicle. A well-established positive control of elevated plus maze, diazepam intraperitoneal (i.p.) injection (1 mg kg^−1^, obtained from DEG Imp. de Produtos Químicos Ltda, Brazil) was also examined in this task. VF injections were given 30 min before the elevated plus maze and 30 min before each training of the behavioral test.

### 2.7. Open-Field Behavior

The open-field exploration was carried out as previously described [16]. The open field was a 50 × 25 cm arena, surrounded by 50 cm high walls, and made of plywood with a frontal glass wall. The floor of the arena was divided into 12 equal squares by black lines. Animals were placed on the left rear quadrant and left to freely explore the arena for 5 min. Latency to start locomotion, crossings of the black lines, rearings performed and the number of fecal pellets were counted. The numbers of crossings and rearings were used as measures of locomotor activity and exploratory behavior, respectively, whereas the latency to start locomotion and the number of fecal pellets were used as indicators of anxiety. After 24 hours, animals were left to explore the apparatus again for another 5 min, and the same measures were recorded to evaluate habituation memory to the open field.

### 2.8. The Elevated Plus-Maze Test

The elevated plus maze used in this study was modified from Lister [17]. The apparatus, elevated 45 cm from the floor, consisted of two open arms (30 × 6 cm) opposite to one another and crossed at right angles by two enclosed arms (30 × 6 × 15 cm) with an open roof. Anxiolytic compounds selectively increase the percentage of time spent and/or arm entries in the open arms; in contrast, anxiogenic compounds selectively decrease the percentage of time spent and/or arm entries in the open arms. The number of entries and the total time spent in each of the two arm types were taken during a 5-min test period after the mice had been placed in the center of the maze 30 min following VF, diazepam or vehicle administration.

### 2.9. Novel Object Recognition

The novel object recognition task was performed as previously described [16]. Object recognition training and test trials took place in the same arena used for the open field. After 24 hours of a 5-min arena habituation session, the mice were trained in the novel object recognition task. Training was conducted by placing individual animals for 5 min into the arena, in which two identical objects (objects A1 and A2; Lego Duplo toys) were positioned in two adjacent corners, 10 cm from the walls. In a long-term memory retention test given 24 hours after training, the same mice explored the field for 5 min in the presence of familiar object A1 and a novel object B. All objects presented similar textures and sizes, but distinctive colors and shapes. Exploration was defined as sniffing or touching the object with the nose and/or forepaws. The exploratory preference was defined as the percentage of the total exploration time that the animal spent investigating object A2 (in the training) or the novel object.

### 2.10. Inhibitory Avoidance

The step-down inhibitory avoidance apparatus and procedures were described in previous studies [16]. The inhibitory avoidance training box was a 50 × 25 × 25 cm acrylic box whose floor consisted of parallel stainless-steel bars. A platform (10 × 10 × 2 cm) was placed on the center of the floor. In the training trial, animals were placed on the platform, and their latency to step-down on the grid with all four paws was recorded. Immediately after stepping down on the grid, animals were given a 0.6 mA/3 s footshock. In the retention test session carried out 24 hours after training, no footshocks were given on test and a ceiling of 180 s was imposed in the test latency.

### 2.11. Statistical Analysis

Open field, habituation, elevated plus maze and novel object recognition data were expressed as mean ± standard error. Data for inhibitory avoidance were expressed as median + interquartile range of step-down latencies. Comparisons among groups were performed using one-way analysis of variance (ANOVA) followed by LSD (parametric data) or Kruskal-Wallis analysis of variance followed by Mann-Whitney *U* (non-parametric data) tests when necessary. Comparisons between behavioral trials within the same group (comparisons between open-field behavior session and habituation session in the open-field test, comparisons between training and test sessions in the novel object recognition and in the inhibitory avoidance) were done by Wilcoxon test. *P*-values of <.05 were considered to indicate statistical significance. Statistical analyses were performed using the statistical software package SPSS.

## 3. Results

### 3.1. Open-Field Behavior and Open-Field Habituation

**Figure 1:**
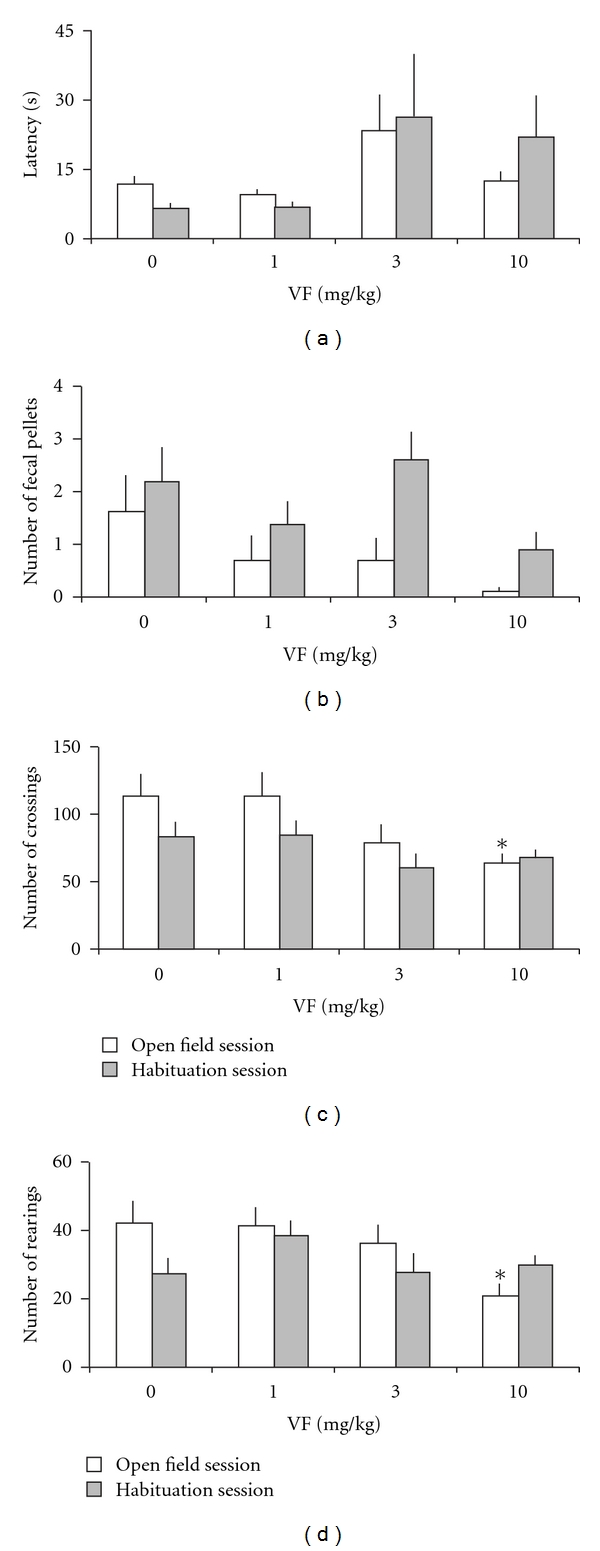
Open-field behavior and habituation in mice treated with a systemic administration of VF (1, 3 or 10 mg kg^−1^) of *V. glechomifolia* 30 min before the first open-field exploration session. Animals were left to freely explore the arena for 5 min day^−1^ during 2 days. Data are mean ± SEM. (a) Latency to start locomotion (s), (b) number of fecal pellets, (c) number of crossings and (d) number of rearings. *n* = 10 animals per group. *P < .05, significant difference from the control group.

The results showed that there were no significant differences among groups in the latency to start locomotion in open-field sessions (*F*(3,36) = 2.35, *P* =  .09; Figure 1(a)), or number of fecal pellets (*F*(3,36) = 1.65, *P* =  .20; Figure 1(b)). However, mice treated with VF at 10 mg kg^−1^ showed significantly lower numbers of crossings (*F*(3,36) = 2.95, *P* =  .046; Figure 1(c)) and rearings (*F*(3,36) = 3.09, *P* =  .039; Figure 1(d)), which indicate alterations in locomotion and reduced exploratory behavior, compared with the control animals. 

Results for open-field habituation session, 24 hours after the first open-field exploration session, are shown in Figure 1. There were no significant differences among groups in the latency to start locomotion (*F*(3,36) = 1.57, *P* =  .21;  Figure 1(a)), number of fecal pellets (*F*(3,36) = 2.32, *P* =  .09; Figure 1(b)), number of crossings (*F*(3,36) = 1.43, *P* =  .25; Figure 1(c)) or number of rearings (*F*(3,36) = 1.23, *P* =  .31; Figure 1(d)), indicating no alterations in locomotion and reduced exploratory behavior in the habituation session, compared with the control animals.

For addressing the habituation process of the open-field experiment (Figure 1) in a more specific way, Wilcoxon tests were applied between sessions of the same treatment. These tests showed the expected significant decrease in the number of rearings during habituation session in the control mice treated with vehicle (*P* =  .008). This profile was maintained in mice that received 3 mg kg^−1^ of VF (*P* =  .038). However, mice treated with 1 mg kg^−1^ of VF showed no difference in the rearings (*P* =  .767), whereas those treated with VF 10 mg kg^−1^ (*P* =  .037) displayed an increased number of rearings during the habituation session, indicating an impairment in the habituation process. Control mice also showed the expected significant decrease in the latency to start locomotion (*P* =  .036), a response that was not observed in any of the VF concentrations. The highest doses of VF (3 and 10 mg kg^−1^) caused mice to produce more fecal pellets (*P* =  .035 and  .039, resp.), indicating increased anxiety in the habituation session (24 hours after VF administration).

### 3.2. The Elevated Plus-Maze Test

**Figure 2:**
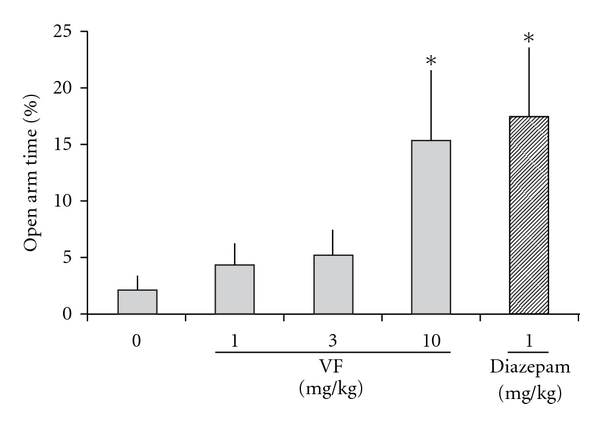
Elevated plus-maze behavior in mice treated with a systemic administration of VF (1, 3 or 10 mg kg^−1^) of *V. glechomifolia* 30 min before behavioral testing. Animals were left to freely explore the apparatus for 5 min. The following parameter is shown: percentage open-arm time (percentage of time spent in open arms with respect to total time spent in the arms). Data are mean ± SEM. *n* = 10 animals per group. *P < .05, significant difference from the control group.

Mice treated with 10 mg kg^−1^ VF or diazepam at 1 mg kg^−1^ showed a significant increase in the percentage of time spent in the open arms when compared with control mice (*F*(3,36) = 2.729, *P* =  .032 and  .014, resp.; Figure 2). In addition, animals treated with diazepam also showed increased total number of arm entries (*F*(3,36) = 5.31, *P* =  .001), whereas the VF treatments did not affect this exploratory behavior significantly (VF 1, 3 and 10 mg kg^−1^; *P* =  .219,  .818 and  .235, resp.). There was no significant difference among VF treatments in the number of open-arms entries on the elevated plus-maze test (*F*(3,36) = 0.315, *P* =  .814). 

### 3.3. Novel Object Recognition

**Figure 3:**
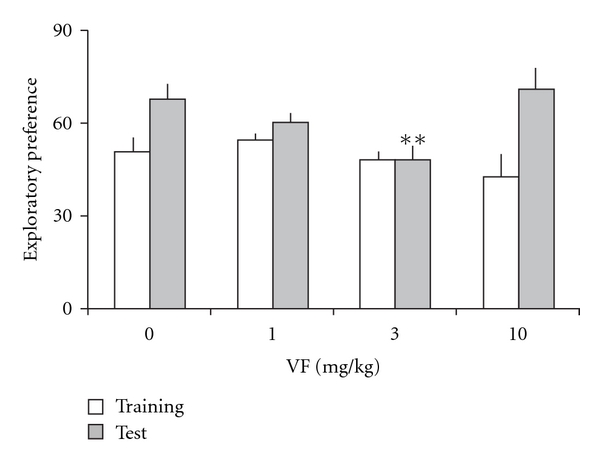
Novel object recognition memory in mice treated with a systemic administration of VF (1, 3 or 10 mg kg^−1^) of *V. glechomifolia* 30 min before the training. Memory retention was tested 24 hours after training. Data are mean ± SEM exploratory preferences during training (light columns) or test (dark columns) trials. Exploratory preference was defined as percentage time exploring object A2 during training or percentage time exploring the novel object B during test trials. *n* = 8 animals per group. **P < .01, significant difference from the control group.

Results for the effects of the VF administration on memory assessed in the novel object recognition task are shown in Figure 3. There were no differences among groups in the total time spent exploring both objects during training (*F*(3,36) = 1.61, *P* =  .21), indicating that all groups showed similar locomotion and motivation during task acquisition. Mean ± SE total exploration time (s) was 80.9 ± 7.2 (control), 87.4 ± 7.9 (VF, 1 mg kg^−1^), 63.3 ± 7.8 (VF, 3 mg kg^−1^), and 70.6 ± 11.2 (VF, 10 mg kg^−1^). There was no significant difference between groups in exploratory preference in the training trial (*F*(3,36) = 1.30, *P* =  .29). Moreover, there was a significant difference among groups in exploratory preference during test (*F*(3,36) = 4.37, *P* =  .01). Further analysis revealed a significant difference between the control group and the group given VF at 3 mg kg^−1^ in long-term recognition memory retention tested 24 hours after training (*P* =  .008). These findings indicate that pretraining systemic administration of VF at the dose of 3 mg kg^−1^ produced an impairment of novel object recognition memory. 

Wilcoxon tests showed a significantly higher novel object exploratory preference in the VF at dose of 10 mg kg^−1^ (*P* =  .017). The vehicle and VF 1 mg kg^−1^ treated group fell short of significance (*P* =  .069 and  .066, resp.) and there was no significant difference among training and test at VF 3 mg kg^−1^-treated group (*P* =  .515).

### 3.4. Inhibitory Avoidance

**Figure 4:**
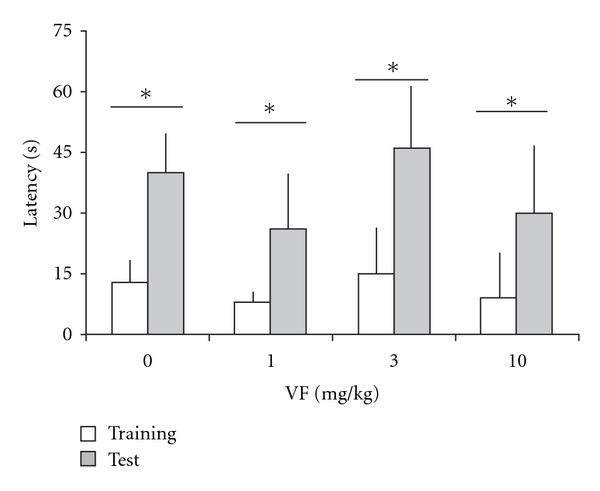
Fear-related memory assessed in an inhibitory avoidance task in mice treated with a systemic administration of VF (1, 3 or 10 mg kg^−1^) of *V. glechomifolia* 30 min before training. Memory retention was tested 24 h after training. Data are median + interquartile range. Latencies to step-down (s) of training (light columns) or test (dark columns). *n* = 9 animals per group. There were no significant differences among groups, either in the training trial or in the test. Asterisks indicate groups showing significant training-test differences (Wilcoxon test, *P* <  .05).

Results for inhibitory avoidance are shown in Figure 4. In all groups, there were significant training-test differences (Wilcoxon test, *P* <  .05). There were no significant differences among groups in step-down latencies in the training trial (*H* = 1.34, *df* = 3, *P* =  .72; mean ± SEM overall training trial step-down latencies was 14.69 ± 1.65 s). In addition, there was no significant difference between groups in long-term memory retention carried out 24 h after training when compared with the control group (*H* = 0.13, *df* = 3, *P* =  .99). The results indicate that VF did not affect inhibitory avoidance memory. 

## 4. Discussion

*Valeriana* sp. contain several compounds including essential oils, terpenoids and small amounts of flavonoids, alkaloids and minerals [2, 5, 6]. Previous studies have reported the effect of higher polarity extracts (such as hydroalcoholic extracts) on the central nervous system [4, 6]. Studies with non-humans tend to support valerian as a central nervous system depressant [18]. Neurobiological mechanisms have been postulated to mediate its sedative and hypnotic effects, including binding studies for gamma-amino butyric acid (GABA) [18, 19], serotononergic [8, 20], dopaminergic and noradrenergic [18] and A1 adenosine [18, 21] receptors effects.

Although valerian is used traditionally as a mild sedative, research is sparse, and studies differ greatly with respect to design, measures and preparations used [22]. The role of valepotriates is considered somewhat controversial [6]. In this study, the extract containing 96% of valepotriates of *V. glechomifolia* at 10 mg kg^−1^ was effective in reducing locomotion and exploratory behavior during open-field exploration in mice, which is indicative of sedative properties. This concentration also increased the time spent on the open arms, an indicative of anxiolytic property in the elevated plus-maze test, a well-established rodent model of anxiety [23]. However, unlike diazepam (1 mg kg^−1^), VF (10 mg kg^−1^) did not alter open-arm entries and total arm entries, but affected the time in closed arms. The effect is dose dependent, with lower doses of VF having no influence. Moreover, this dose did not affect the memory tests, compared with control animals. One possible interpretation for the lack of effect of VF 10 mg kg^−1^ on the number of arm entries is partial locomotion impairment as observed in Figure 1(c). However, low doses of diazepam are known to induce increase in locomotion and number of arm entries [23, 24], which may also have contributed to this result.

Neither object exploration during training in a novel object recognition task nor inhibitory avoidance performance was affected by VF, whereas 24 h retention of recognition memory was impaired by a lower dose of VF that did not affect locomotion or exploration. Thus, a lower dose of VF can selectively affect formation of recognition memory without inducing overt non-specific effects on other behavioral parameters.

It is interesting to note that the VF effect that impaired novel object recognition memory at the dose of 3 mg kg^−1^ was not observed at the lower 1 mg kg^−1^ and higher 10 mg kg^−1^ doses. Previous studies evaluating the effects of injections of both memory enhancing [25] and memory impairing [26] drugs on memory show that several treatments produce an inverted-U dose-response curve [27].

This is the first study, to our knowledge, that shows the neuropharmacological profile of an enriched VF from *V. glechomifolia*, and the effect of this valepotriate extract in memory. Obviously, a crude extract may have different activity due to the presence of additional compounds and interactions, which was not evaluated in the present work because the focus was on valepotriates, the major phytochemical components in this species. In addition, valerian-based herbal medicines are usually taken orally, an intake route through which valepotriates are poorly absorbed, even though some of their catabolites, such as baldrinal, may remain available [7]. I.p. injections as employed in the present study may considerably change bioavailability compared with oral intake, which may affect the observed activity. However, i.p. injections were used to provide homogeneous applications and to maximize bioavailability of valepotriates, the phytochemicals evaluated in this first investigation of pharmacological properties of *V. glechomifolia*.

In summary, the present study indicates that systemic administration of VF from *V. glechomifolia* has sedative properties and can induce alterations in recognition memory and in the elevated plus-maze behavior in mice. Further research is required to examine the neurochemical mechanisms involved in the behavioral effects.

## Funding

Brazilian federal research funding organizations: Coordination of Improvement of Higher Education (CAPES - Coordenação de Aperfeiçoamento de Pessoal de Nível Superior) and National Counsel of Technological and Scientific Development (CNPq - Conselho Nacional de Desenvolvimento Científico e Tecnológico).
